# Severe leukopenia in *Staphylococcus aureus*-necrotizing, community-acquired pneumonia: risk factors and impact on survival

**DOI:** 10.1186/1471-2334-13-359

**Published:** 2013-08-01

**Authors:** Nagham Khanafer, Nicolas Sicot, Philippe Vanhems, Oana Dumitrescu, Vanina Meyssonier, Anne Tristan, Michèle Bès, Gérard Lina, François Vandenesch, Yves Gillet, Jérôme Etienne

**Affiliations:** 1 Université Lyon1, CNRS UMR 5558, Villeurbanne, F-69622, France; 2 Hospices Civils de Lyon, Groupement Hospitalier Edouard Herriot, Service d’Hygiène, Épidémiologie et Prévention, Place d’Arsonval, Lyon, F-69437, France; 3 Hospices Civils de Lyon, Centre National de Référence des Staphylocoques, Bron, F-69677, France; 4 Hôpital Pitié Salpêtrière, Paris, F-75013, France; 5 Université Lyon1, INSERM U851, Lyon, F-69008, France; 6 Hospices Civils de Lyon, Hôpital Femme Mère Enfant, Bron, F-69677, France

**Keywords:** Community-acquired pneumonia, Staphylococcus aureus, Panton valentine leukocidin, Leukopenia

## Abstract

**Background:**

Necrotizing pneumonia attributed to Panton-Valentine leukocidin-positive *Staphylococcus aureus* has mainly been reported in otherwise healthy children and young adults, with a high mortality rate. Erythroderma, airway bleeding, and leukopenia have been shown to be predictive of mortality. The objectives of this study were to define the characteristics of patients with severe leukopenia at 48-h hospitalization and to update our data regarding mortality predicting factors in a larger population than we had previously described.

**Methods:**

It was designed as a case-case study nested in a cohort study. A total of 148 cases of community-acquired, necrotizing pneumonia were included. The following data were collected: basic demographic information, medical history, signs and symptoms, radiological findings and laboratory results during the first 48 h of hospitalization. The study population was divided into 2 groups: (1) with severe leukopenia (leukocyte count ≤3,000 leukocytes/mL, n=62) and (2) without severe leukopenia (>3,000 leukocytes/mL, n=86).

**Results:**

Median age was 22 years, and the male-to-female gender ratio was 1.5. The overall in-hospital mortality rate was 41.2%. Death occurred in 75.8% of severe leukopenia cases with median survival time of 4 days, and in 16.3% of cases with leukocyte count >3,000/mL (*P*<0.001). Multivariate analysis indicated that the factors associated with severe leukopenia were influenza-like illness (adjusted odds ratio (aOR) 4.45, 95% CI (95% confidence interval) 1.67-11.88, *P*=0.003), airway bleeding (aOR 4.53, 95% CI 1.85-11.13, *P*=0.001) and age over 30 years (aOR 2.69, 95% CI 1.08-6.68, *P*=0.033). A personal history of furuncles appeared to be protective (OR 0.11, 95% CI 0.01-0.96, *P*=0.046).

**Conclusion:**

*S. aureus-*necrotizing pneumonia is still an extremely severe disease in patients with severe leukopenia. Some factors could distinguish these patients, allowing better initial identification to initiate adapted, rapid administration of appropriate therapy.

## Background

Since the description of necrotizing pneumonia due to Panton-Valentine leukocidin (PVL)-positive *Staphylococcus aureus* by Gillet *et al.* in 2002, numerous cases have been reported worldwide [1-7]. They mainly impacted otherwise healthy children and young adults, with a median age of 14 years. They are attributed to either methicillin-sensitive or -resistant *S. aureus* strains of various genetic backgrounds, but have all PVL genes in common [8,9]. Necrotizing pneumonia is characterized by rapid, extensive, bilateral pneumonia frequently evolving towards acute respiratory distress syndrome (ARDS), despite intensive medical interventions with mechanical ventilation and inotrope support. No specific treatment guidelines have been published so far, but the addition of toxin-suppressing antibiotics, such as clindamycin, linezolid and rifampicin, has been suggested [10-12].

The overall mortality rate ranges from 30 to 75% of cases [3,13]. The fatal outcome is rapid, with median survival time between 4 to 10 days from the onset of symptoms [1,4,12]. It is associated with classic severity factors, such as the need for mechanical ventilation or inotrope support, and the onset of ARDS. Erythroderma, airway bleeding, and leukopenia have been shown to be predictive of mortality. Gillet *et al.* reported significant differences, by multivariate analysis, in median leukocyte count between patients who survived and those who did not. The survival rate was <10% when the leukocyte count was <3,000 leukocytes/mL. The leukopenia observed in patients could reflect PVL cytotoxicity, demonstrated in vitro on human neutrophils [14,15].

As patients with leukopenia face a high risk of mortality, better initial recognition of these severe cases would allow rapid administration of appropriate treatment. In this study, a series of 148 cases of *S. aureus-*necrotizing pneumonia was analyzed. Our aim was to define the characteristics of patients with severe leukopenia at 48-h hospitalization and to update our data regarding mortality predicting factors in a larger population than we had previously described [13].

## Methods

### Data source

Since 1986, the *French National Reference Centre of Staphylococci* (Lyon, France) has collected 161 case reports of documented pneumonia caused by *S. aureus* strains carrying PVL genes (*lukS*-PV*—lukF*-PV). Informed consent was waived because data were extracted from the surveillance database. According to French law, a study like this one does not require ethics committee approval because it is observational and based on a surveillance database approved under national regulations. The protocol design was approved by the hospital’s institutional review board (*Comité National Informatique et Liberté*).

### Definitions

Pneumonia was defined by signs and symptoms of lower respiratory tract infection (e.g., cough, expectoration, and chest pain) and pulmonary infiltrates on chest X Ray reviewed by a radiologist, that were not attributable to other causes, but coinciding with *S. aureus* isolation by at least 1 of the following procedures: (1) pleural effusion or lung abscess; (2) broncho-alveolar lavage fluid culture (10^4^ CFU/mL), Wimberley brushing (10^3^ CFU/mL), or protected tracheal aspiration (10^3^ CFU/mL); and (3) blood culture revealing *S. aureus* as the sole potential pathogen. Cases with respiratory symptoms starting at least 48 h after hospitalization were classified as nosocomial and were excluded from the study. Leukopenia was defined as severe if median leukocyte count was < 3,000 leukocytes/mL within the first 48 h after hospital admission.

### Microbiological studies

*S. aureus* isolates were tested for antimicrobial susceptibility and toxin production. Testing of isolates for antimicrobial susceptibility by broth microdilution was undertaken according to the interpretive criteria of the Clinical and Laboratory Standards Institute (formerly the National Committee for Clinical Laboratory Standards). The following antimicrobial agents were used: penicillin, oxacillin, kanamycin, tobramycin, gentamicin, erythromycin, clindamycin, tetracycline, ofloxacin, fusidic acid, rifampicin, vancomycin, teicoplanin, fosfomycin, trimethoprim-sulfamethoxazole, and linezolid. Gene sequences encoding superantigens (enterotoxins A-E, G-I, and toxic shock syndrome toxin), PVL and *mec*A gene, which codes for methicillin resistance, were detected by PCR, as described elsewhere [16]. Only cases caused by PVL-positive *S. aureus* strains were included.

### Study design and population

Designed as a case-case study nested in a cohort study, analyses were restricted to 148 cases where all clinical and biological data were available.

### Data collection

The following data were collected by a standardized form and comprised basic demographic information, medical history (including risk factors for infection and history of personal or familial abscesses or furuncles), signs and symptoms, radiological findings and laboratory results during the first 48 h of hospitalization. Severity was rated by Pediatric Risk of Mortality (PRISM) 3 scores for patients <18 years and the Simplified Acute Physiology Score (SAPS) II for patients ≥18 years, when available. Some biological and radiological data were missing because of death shortly after admission to hospital.

### Statistical analysis

Categorical variables were compared by the Chi-square or Fisher’s exact test, and continuous variables, by Student’s t-test or Mann–Whitney tests. Survival probability according to median leukocyte count, was estimated by the Kaplan-Meier method. Initially the cases were divided into 3 groups: 0–3,000; 3,000-10,000 and >10,000 leukocytes/mL but we merged the data of the last two groups since no significant difference was found for variables included in the final multivariate regression model (data not shown in this paper). The ROC analysis showed a 84% sensitivity for a cut-off of 3000 leukocytes/mL.

Baseline was the day of admission to hospital because of pneumonia, and patients who survived were censored at hospital discharge. When patients died within 24 h after admission, the observation period was rounded to 1 day. Survival distributions were compared by the log-rank test. Variables independently associated with survival were identified with a Cox regression model based on hazard ratios with 95% confidence interval (95% CI).

Variables associated with severe leukopenia were tested by the multivariate logistic regression model. When *P* values of variables described in the first table, were <0.20 in univariate analysis, they were submitted to the multivariate model. Variables in multivariate analysis were subjected to the forced entry procedure, with stepwise and backward elimination, using *P* values of 0.1 as criteria for inclusion and elimination of risk variables based on best subset logistic regression with Chi-square score fit criteria. The Hosmer-Lemeshow test assessed the model’s goodness-of-fit. Adjusted odds ratios (aOR) and corresponding 95% CI were calculated. For all tests performed, 2-tailed *P* values < 0.05 were regarded as denoting statistical significance. Analyses were performed with SPSS 17.0 software (SPSS Inc., Chicago, IL, USA).

## Results

From 1986 through 2010, 161 cases of community-acquired, necrotizing pneumonia were collected and documented; 13 case reports were excluded because of missing data concerning leukocytes count.

### Demographic conditions and medical history

In total, 148 cases of community-acquired necrotizing pneumonia were included. Median age was 22 years (interquartile range [IQR] 3.0-43.7) and the male-to-female gender ratio was 1.5 (88 males and 60 females). Smoking was reported in 15.5% patients. Common risk factors for staphylococcal infection, such as diabetes, steroid therapy, and immunosuppressive treatment, were noted for 6.1%, 6.8% and 5.4% of patients, respectively. Among the 126 patients for whom data were available, 10.3% had a personal history of furuncles or skin abscess.

The median duration of symptoms prior to hospitalization was 3.0 days (IQR 2.0-5.0 days), with preceding influenza-like syndrome in 61.7% (87 of 141), and pre-existing skin and soft tissue infection (SSTI) in 22.3% (33 of 143).

### Clinical and biological data

The clinical course during the first 48 h after hospital admission was usually severe, with 63.5% of patients requiring mechanical ventilation. The most remarkable clinical feature was airway bleeding, which occurred in 40.0% of patients. *S. aureus* was recovered from blood culture in 60.8%, in pleural fluid from 40.5%, and in tracheal aspirates from 12.8% of patients.

Median minimal leukocyte count during the first 48 h of hospitalization was 4,700 leukocytes/mL (IQR 1,300-12,800 leukocytes/mL), and 41.9% had leukocyte count ≤3,000 leukocytes/mL. The minimal platelet count was also low, with a median of 168,000 platelets/mL (IQR 88,000-275,000 platelets/mL).

The overall in-hospital mortality rate was 41.2%. 98.4% of deaths were attributed to *S. aureus* and were secondary to refractory shock and/or respiratory failure.

#### Factors associated with severe leukopenia

The study population was divided into 2 groups: (1) with severe leukopenia (leukocyte count ≤3,000 leukocytes/mL, n=62) and (2) without severe leukopenia (>3,000 leukocytes/mL, n=86).

The group with severe leukopenia was characterized by a significantly higher rate of female patients (*P*=0.008) (Table 1). Prior influenza-like illness (ILI: 79.3% vs. 49.4%, *P*<0.001) and airway bleeding (62.9% vs. 23.3% (*P*<0.001) were also associated with severe leukopenia. Conversely, a personal history of furuncles or skin abscess (*P*=0.001) and SSTI at admission (*P*=0.002) were more frequent in the group without severe leukopenia. The group with severe leukopenia presented ARDS onset more frequently and needed artificial ventilation or inotrope support (*P*<0.001).

**Table 1:** **Characteristics of severe and non-severe leukopenia in a French cohort of 148 patients with *S. aureus-*necrotizing pneumonia, between 1986 and 2010**

**Variables**	**Group 1**	**Group 2**	***P *value**
	**(≤3.000 leukocytes/mL) n=62**	**(>3 000 leukocytes/mL) n=86**	
**Demographic information**			
**Age**			
0-30	32 (51.6)	55 (64.0)	0.132
>30	30 (48.4)	31 (36.0)	
Gender, M:F (ratio)	29:33 (46.8)	59:27 (68.6)	0.008
**Medical history**			
Smoking	7 (12.5)	16 (19.5)	0.278
Alcohol	1 (1.7)	3 (3.7)	0.479
Steroid treatment	2 (3.3)	7 (8.3)	0.222
Respiratory failure	0 (0)	3 (3.5)	0.138
Personal history of furuncles or skin abscess	1 (2.0)	12 (16.0)	0.011
**Before hospital admission**			
Period between onset of symptoms and admission^a^	3 (2-4)	3 (2-5)	0.684
Influenza-like illness	46 (79.3)	41(49.4)	<0.001
SSTI^b^	6 (9.7)	27 (31.4)	0.002
**Clinical features within the first 48 h of hospitalization**			
PRISM score^a, b^	27 (20-35)	6 (4-11)	<0.001
SAPS II score^a, b^	67 (39-80)	28 (15-61.8)	<0.001
Platelet count/mL	92,000 (46,500-176,500)	204,000 (145,000-370,000)	<0.001
Fever, temperature >39°C	46 (64.2)	70 (81.4)	0.294
Generalized rash 24 h	5 (8.3)	6(7.0)	0.760
Airway hemorrhage	39 (62.9)	20 (23.3)	<0.001
Pleural effusion	16 (27.1)	49 (57.6)	<0.001
Radiological consolidation			0.001
Unilobar	6 (9.8)	30 (34.9)	
Multilobar	48 (78.7)	44 (51.2)	
ARDS^b^	47 (77.0)	15 (18.1)	<0.001
Methicillin resistance (*mec*Agene)	15 (24.2)	20 (23.3)	0.895
**Treatment and outcome**			
Mechanical ventilation	57 (91.9)	37 (43.0)	<0.001
Inotrope drugs	52 (91.2)	21 (26.3)	<0.001
Time to death^a^	2 (1-6)	2 (1-2.75)	0.216
Appropriate antibiotics introduced in first 24 h of h hospitalization	46 (75.4)	65 (75.6)	0.161
Mortality	47 (75.8)	14 (16.3)	<0.001
Percentage of deaths attributed to *S. aureus* infection	47 (100)	13 (92.9)	0.065

^a^Continuous variables are indicated as median, first and third quartiles and categorical variables as number (%).

^b^SSTI: skin and soft tissue infection, PRISM: Pediatric Risk of Mortality; SAPS II: Simplified Acute Physiology Score; ARDS: acute respiratory distress syndrome.

Multivariate analysis indicated that ILI, airway bleeding and age over 30 years were independent factors associated with severe leukopenia (Table 2). A personal history of furuncles appeared to be protective.

**Table 2:** **Factors associated with severe leukopenia among patients with Panton-Valentine leukocidin-positive *Staphylococcus aureus*-associated necrotizing pneumonia**

**Variables**^**a**^	**Adjusted odds ratios (95% CI)**	***P *value**
Previous influenza-like illness	4.45 (1.67-11.88)	0.003
Age >30 years	2.69 (1.08-6.68)	0.033
Airway hemorrhage	4.53 (1.85-11.13)	0.001
Personal history of furuncles	0.11 (0.01-0.96)	0.046

^**a**^Variables were adjusted to gender, mechanical ventilation, platelet count, pleural effusion, and time elapsed between onset of symptoms and hospitalization.

### Survival analysis

**Figure 1:**
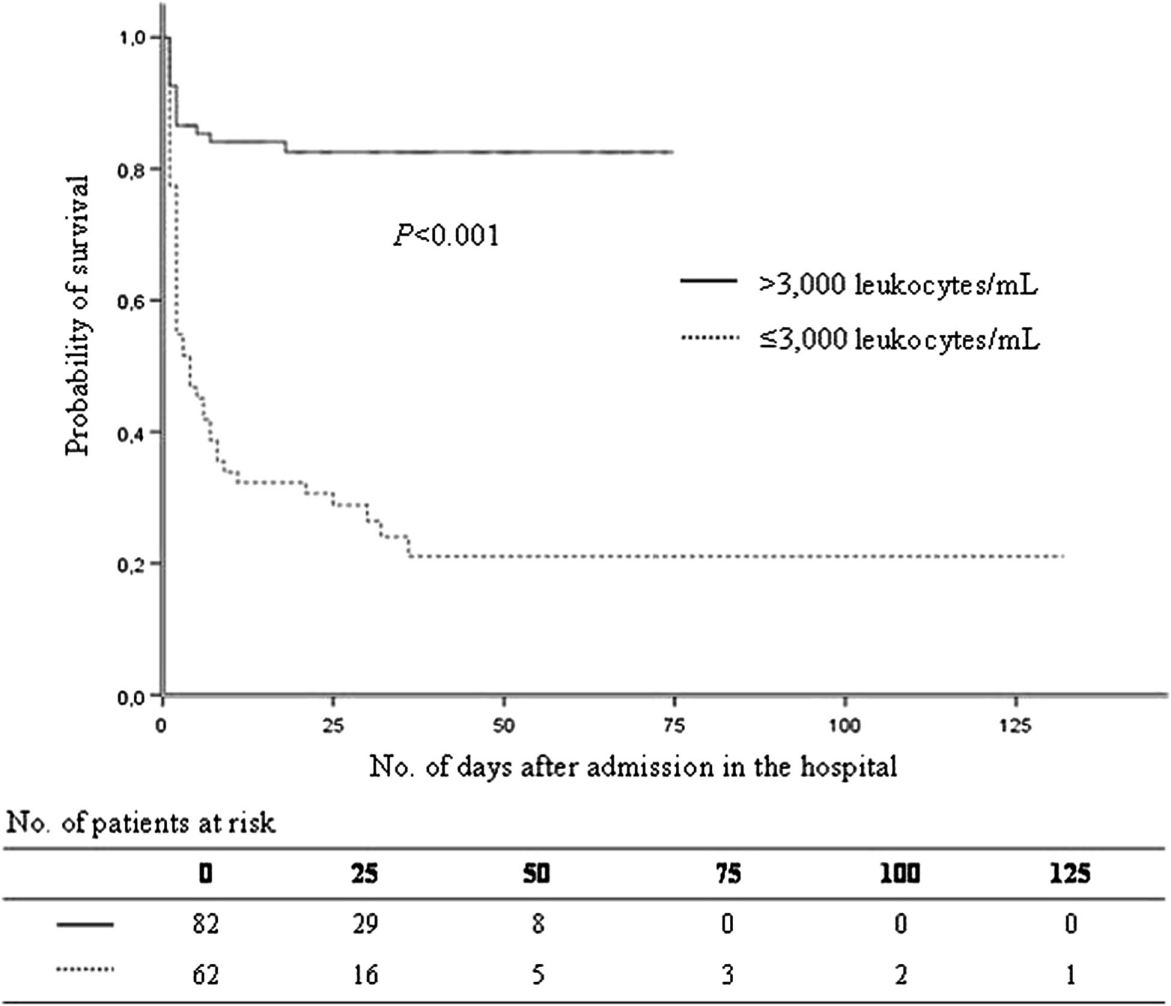
**Probability of survival among patients with Panton-Valentine leukocidin-positive *Staphylococcus aureus *pneumonia, according to leukocyte count.**

Leukocyte count was negatively correlated with mortality. Death occurred in 75.8% of cases (47 of 62) with severe leukopenia (≤3,000 leukocytes/mL) with median survival time of 4 days (Figure 1). Only 16.3% of cases (14 of 86) with leukocyte count >3,000/mL (*P*<0.001) died. Mortality was 66% in cases with airway bleeding versus 24.7% in those without (*P*<.001). Cox multivariate analysis indicated that the only factors associated with fatal outcome were leukopenia, airway hemorrhage and age (Table 3).

**Table 3:** **Factors associated with death among patients with Panton-Valentine leukocidin-positive *Staphylococcus aureus *pneumonia**

**Variables**	**Adjusted hazard ratio (95% CI)**	***P *value**
Age (per 1-year increase)	1.02 (1.01-1.03)	0.015
Airway hemorrhage	2.05 (1.18-3.59)	0.011
Severe leukopenia (≤3,000 leukocytes/mL)	4.50 (2.38-8.51)	<0.001

Variables were adjusted to gender, mechanical ventilation, personal history of furuncles, platelet count, pleural effusion, and previous influenza-like illness.

## Discussion

In this article, we compared the characteristics of a group of 62 cases of severe leukopenia (≤3,000 leukocytes/mL) with a group of 86 patients without severe leukopenia (>3,000 leukocytes/mL) at 48 h of hospitalization.

The group with severe leukopenia was exemplified by a significantly higher rate of female patients (*P*=0.008), prior ILI (*P*<0.001) and airway bleeding (*P*<0.001). Conversely, a personal history of furuncles or skin abscess (*P*=0.001) and SSTI at admission (*P*=0.002) were more frequent in the group without severe leukopenia. The mortality rate was significantly different between groups (75.8% vs 16.3%, *P*<0.001).

Necrotizing pneumonia largely occurred in young people with a median age of 22 years, only slightly higher than reported earlier in smaller series [1,3,7,13,17]. The increasing of age of included patients might be attibuable to a delayed exposure to the causative organism. This epidemiological trend should be challenged by similar surveys in other countries. This effect could be related to the improvement of the management of this infection.

Our study confirms the association of leukocyte count with disease severity. By multivariate logistic regression, age over 30 years was associated with severe leukopenia (aOR 2.69, *P*=0.033). The reason of this association was not clear. The increasing of age of patients might be attibuable to a delayed exposure to the causative organism. This epidemiological trend should be challenged by similar surveys in other countries. An association between time and lymphocyte count was actually reported in different populations [18,19].

Female gender was associated with severe leukopenia (OR 2.49, *P*=0.008) by univariate analysis but not by multivariate analysis. Females in general mount a more profound immune response after antigenic challenge, and these differences have mainly been attributed to the immunomodulatory effects of sex hormones, despite the lack of human *in vivo* data. Even though we still found some papers reporting an association of neutropenia with female gender. vanEijk *et al*. demonstrated, in an *in vivo* study, that females exert a more pro-inflammatory pattern of cytokine release compared to males during systemic inflammation after the administration of *Escherichia coli* endotoxin [20]. This difference is associated with more leukocyte sequestration in females [20]. Sterling *et al*. reported gender-based differences in host immune responses [21]. In a study analyzing the risk factors for developing neutropenia after mitomycin C administration, multivariate logistic regression showed that female gender was an independent risk factor for neutropenia and the reasons for this association are unknown [22].

The significant association with a previous ILI (aOR 4.45, *P*=0.003) might be related to a particular linkage with influenza virus. Possible epithelial damage, due to viral infection, could promote the pathogenicity of PVL-producing strains with increasing affinity for collagen (I and IV) and laminin [23].

A personal history of furuncles appears to be protective (OR 0.11, 95% CI 0.01-0.96, *P*=0.046). It is conceivable that patients with such a history had previously been exposed to PVL and had developed a degree of protective immunity [24]. Another hypothesis could explain this association: after bacteria reach the lungs through the bloodstream, the necrotizing effect is less serious.

The present investigation has some limitations. First, case reporting to the Reference Center was unsolicited and may not reflect accurate epidemiology and disease severity. Severe and dramatic cases among previously-healthy young people are more likely to be reported. A bias of notification would not be excluded. This would be caused by some missing data possibly not declared after 2000 due to the dispersion of laboratories dealing with PVL gene detection.

Moreover the median age was slightly higher than had been previously reported which could be related to the introduction of several novelties in the treatment of severely ill patients since the identification of this pathology.

Another limitation concerns clinical data collection: they were missing or incomplete in 8% of cases. Analysis included only 148 cases for which microbiological and demographic information were available; 13 cases were excluded from further analysis because they lacked data on leukocyte count. Comparisons between the 13 excluded cases and the 148 cases analyzed revealed no significant differences.

Leukopenia may be simply considered as a reflection of disease severity. This is in agreement with in vitro data showing that PVL induces both apoptosis and necrosis in human leukocytes [25]. The time period between the onset of symptoms and hospitalization could be important to reach a certain level of severity and impaired leukocyte count, but it was not different between both groups. On the other hand, multivariate logistic regression was adjusted to the time factor. Furthermore we noticed that factors associated with severe leukopenia were quite different from those associated with mortality.

Based on our results, we can suggest two syndromes of necrotizing pneumonia: 1) those that are mainly related to ILI and direct inoculation of staphylococci to damaged respiratory epithelium, airway hemorrhage, severe leukopenia and death; and 2) those that are mainly related to hematogenous spread from SSTI, less airway hemorrhage, without leukopenia and improved survival. In conclusion, our study emphasizes that *S. aureus*-necrotizing pneumonia is still an extremely severe disease. We found that some factors could distinguish patients with severe leukopenia from those without leukopenia.

Leukocytes count is promptly available at admission to the hospital and can be easily used to asses the severity of disease as suggested by this paper. The impact of this marker, on patient management, need to be clarified. Empiric therapy should include coverage for *S. aureus* as soon as possible, without waiting for the bacteriological results.

Clinical data indicate that neutralizing toxin production improves the outcome [26,27]. The toxin can be blocked by combining a toxin-suppressing agent (e.g., clindamycin, linezolid or rifampin) with bactericidal antibiotics acting on the cell wall [10].

## Conclusions

*S. aureus-*necrotizing pneumonia is still an extremely severe disease in patients with severe leukopenia. Some factors like leukocytes count could distinguish these patients, allowing better initial identification to initiate adapted, rapid administration of appropriate therapy. This paper could be regarded as a preliminary work. Experts are invited to work on a widely accepted score, validate a score including leukocytes count, to predict the severity of this disease.

## Competing interests

The authors declare that they have no competing interests.

## Authors’ contributions

JE, PV, FV, YG, OD, VM, AT, MB and GL conceived of and designed the study; NK, NS, and PV performed the data analysis; and NK, NS, and JE wrote the paper. All authors read and approved the final manuscript.

## Pre-publication history

The pre-publication history for this paper can be accessed here:

http://www.biomedcentral.com/1471-2334/13/359/prepub

## References

[B1] Gillet Y Issartel B Vanhems P Fournet JC Lina G Bes M Vandenesch F Piemont Y Brousse N Floret D Etienne J Association between Staphylococcus aureus strains carrying gene for Panton-Valentine leukocidin and highly lethal necrotising pneumonia in young immunocompetent patients Lancet 2002 359 9308 753 759 10.1016/S0140-6736(02)07877-7 11888586

[B2] Boussaud V Parrot A Mayaud C Wislez M Antoine M Picard C Delisle F Etienne J Cadranel J Life-threatening hemoptysis in adults with community-acquired pneumonia due to Panton-Valentine leukocidin-secreting Staphylococcus aureus Intensive Care Med 2003 29 10 1840 1843 10.1007/s00134-003-1918-5 12904849 PMC7095030

[B3] Francis JS Doherty MC Lopatin U Johnston CP Sinha G Ross T Cai M Hansel NN Perl T Ticehurst JR Carroll K Thomas DL Nuermberger E Bartlett JG Severe community-onset pneumonia in healthy adults caused by methicillin-resistant Staphylococcus aureus carrying the Panton-Valentine leukocidin genes Clin Infect Dis 2005 40 1 100 107 10.1086/427148 15614698

[B4] Hageman JC Uyeki TM Francis JS Jernigan DB Wheeler JG Bridges CB Barenkamp SJ Sievert DM Srinivasan A Doherty MC McDougal LK Killgore GE Lopatin UA Coffman R MacDonald JK McAllister SK Fosheim GE Patel JB McDonald LC Severe community-acquired pneumonia due to Staphylococcus aureus, 2003–04 influenza season Emerg Infect Dis 2006 12 6 894 899 10.3201/eid1206.051141 16707043 PMC3373026

[B5] Klein JL Petrovic Z Treacher D Edgeworth J Severe community-acquired pneumonia caused by Panton-Valentine leukocidin-positive Staphylococcus aureus: first reported case in the United Kingdom Intensive Care Med 2003 29 8 1399 10.1007/s00134-003-1844-6 12783162 PMC7095159

[B6] Miyashita T Shimamoto Y Nishiya H Koshibu Y Sugiyama H Ono Y Satoh T Haraoka H Nakano J Ohta K Sato T Morinaga N Noda M Destructive pulmonary embolism in a patient with community-acquired staphylococcal bacteremia J Infect Chemother 2002 8 1 99 102 11957128 10.1007/s101560200014

[B7] Naimi TS LeDell KH Como-Sabetti K Borchardt SM Boxrud DJ Etienne J Johnson SK Vandenesch F Fridkin S O’Boyle C Danila RN Lynfield R Comparison of community- and health care-associated methicillin-resistant Staphylococcus aureus infection JAMA 2003 290 22 2976 2984 10.1001/jama.290.22.2976 14665659

[B8] Davis SL Perri MB Donabedian SM Manierski C Singh A Vager D Haque NZ Speirs K Muder RR Robinson-Dunn B Hayden MK Zervos MJ Epidemiology and outcomes of community-associated methicillin-resistant Staphylococcus aureus infection J Clin Microbiol 2007 45 6 1705 1711 10.1128/JCM.02311-06 17392441 PMC1933099

[B9] Gonzalez BE Hulten KG Dishop MK Lamberth LB Hammerman WA Mason EO Jr Kaplan SL Pulmonary manifestations in children with invasive community-acquired Staphylococcus aureus infection Clin Infect Dis 2005 41 5 583 590 10.1086/432475 16080077

[B10] Dumitrescu O Badiou C Bes M Reverdy ME Vandenesch F Etienne J Lina G Effect of antibiotics, alone and in combination, on Panton-Valentine leukocidin production by a Staphylococcus aureus reference strain Clin Microbiol Infect 2008 14 4 384 388 10.1111/j.1469-0691.2007.01947.x 18261123

[B11] Stevens DL Ma Y Salmi DB McIndoo E Wallace RJ Bryant AE Impact of antibiotics on expression of virulence-associated exotoxin genes in methicillin-sensitive and methicillin-resistant Staphylococcus aureus J Infect Dis 2007 195 2 202 211 10.1086/510396 17191165

[B12] Talan DA Moran GJ Abrahamian FM Severe sepsis and septic shock in the emergency department Infect Dis Clin North Am 2008 22 1 1 31 v 10.1016/j.idc.2007.09.005 18295681

[B13] Gillet Y Vanhems P Lina G Bes M Vandenesch F Floret D Etienne J Factors predicting mortality in necrotizing community-acquired pneumonia caused by Staphylococcus aureus containing Panton-Valentine leukocidin Clin Infect Dis 2007 45 3 315 321 10.1086/519263 17599308

[B14] Diep BA Chan L Tattevin P Kajikawa O Martin TR Basuino L Mai TT Marbach H Braughton KR Whitney AR Gardner DJ Fan X Tseng CW Liu GY Badiou C Etienne J Lina G Matthay MA DeLeo FR Chambers HF Polymorphonuclear leukocytes mediate Staphylococcus aureus Panton-Valentine leukocidin-induced lung inflammation and injury Proc Natl Acad Sci USA 2010 107 12 5587 5592 10.1073/pnas.0912403107 20231457 PMC2851770

[B15] Loffler B Hussain M Grundmeier M Bruck M Holzinger D Varga G Roth J Kahl BC Proctor RA Peters G Staphylococcus aureus panton-valentine leukocidin is a very potent cytotoxic factor for human neutrophils PLoS Pathog 2010 6 1 e1000715 10.1371/journal.ppat.1000715 20072612 PMC2798753

[B16] Murakami Y Diagnosis of a predisposition of retinoblastoma at the DNA level Gan To Kagaku Ryoho 1991 18 1 44 50 1670986

[B17] Klein E Smith DL Laxminarayan R Community-associated methicillin-resistant Staphylococcus aureus in outpatients, United States, 1999–2006 Emerg Infect Dis 2009 15 12 1925 1930 10.3201/eid1512.081341 19961671 PMC3044510

[B18] Kuranda K Vargaftig J de la Rochere P Dosquet C Charron D Bardin F Tonnelle C Bonnet D Goodhardt M Age-related changes in human hematopoietic stem/progenitor cells Aging Cell 2011 10 3 542 546 10.1111/j.1474-9726.2011.00675.x 21418508

[B19] Bartman MT Kaidarova Z Hirschkorn D Sacher RA Fridey J Garratty G Gibble J Smith JW Newman B Yeo AE Murphy EL HTLV Outcomes Study (HOST) Investigators: Long-term increases in lymphocytes and platelets in human T-lymphotropic virus type II infection Blood 2008 112 10 3995 4002 10.1182/blood-2008-05-155960 18755983 PMC2581993

[B20] van Eijk LT Dorresteijn MJ Smits P van der Hoeven JG Netea MG Pickkers P Gender differences in the innate immune response and vascular reactivity following the administration of endotoxin to human volunteers Crit Care Med 2007 35 6 1464 1469 10.1097/01.CCM.0000266534.14262.E8 17452928

[B21] Sterling TR Pisell-Noland T Perez JL Astemborski J McGriff JR Nutting L Hoover DR Vlahov D Bollinger RC Sex-based differences in T lymphocyte responses in HIV-1-seropositive individuals J Infect Dis 2005 191 6 881 885 10.1086/427827 15717262

[B22] Lambert LA Armstrong TS Lee JJ Liu S Katz MH Eng C Wolff RA Tortorice ML Tansey P Gonzalez-Moreno S Lambert DH Mansfield PF Incidence, risk factors, and impact of severe neutropenia after hyperthermic intraperitoneal mitomycin C Ann Surg Oncol 2009 16 8 2181 2187 10.1245/s10434-009-0523-4 19475451 PMC2711905

[B23] de Bentzmann S Tristan A Etienne J Brousse N Vandenesch F Lina G Staphylococcus aureus isolates associated with necrotizing pneumonia bind to basement membrane type I and IV collagens and laminin J Infect Dis 2004 190 8 1506 1515 10.1086/424521 15378445

[B24] Rasigade JP Sicot N Laurent F Lina G Vandenesch F Etienne J A history of Panton-Valentine leukocidin (PVL)-associated infection protects against death in PVL-associated pneumonia Vaccine 2011 29 25 4185 4186 10.1016/j.vaccine.2011.04.033 21527300

[B25] Genestier AL Michallet MC Prevost G Bellot G Chalabreysse L Peyrol S Thivolet F Etienne J Lina G Vallette FM Vandenesch F Genestier L Staphylococcus aureus Panton-Valentine leukocidin directly targets mitochondria and induces Bax-independent apoptosis of human neutrophils J Clin Invest 2005 115 11 3117 3127 10.1172/JCI22684 16276417 PMC1265849

[B26] Lobo LJ Reed KD Wunderink RG Expanded clinical presentation of community-acquired methicillin-resistant Staphylococcus aureus pneumonia Chest 2010 138 1 130 136 20173050 10.1378/chest.09-1562

[B27] Rouzic N Janvier F Libert N Javouhey E Lina G Nizou JY Pasquier P Stamm D Brinquin L Pelletier C Vandenesch F Floret D Etienne J Gillet Y Prompt and successful toxin-targeting treatment of three patients with necrotizing pneumonia due to Staphylococcus aureus strains carrying the Panton-Valentine leukocidin genes J Clin Microbiol 2010 48 5 1952 1955 10.1128/JCM.01892-09 20129956 PMC2863892

